# Predictors for thromboembolism in patients with cholangiocarcinoma

**DOI:** 10.1007/s00432-021-03794-1

**Published:** 2021-09-09

**Authors:** Christian Pfrepper, Maren Knödler, Ruth Maria Schorling, Daniel Seehofer, Sirak Petros, Florian Lordick

**Affiliations:** 1 Department of Hematology, Cellular Therapy and Hemostaseology, Division of Hemostaseology, University of Leipzig Medical Center, Liebigstr. 20, 04103 Leipzig, Germany; 2 Department of Medicine (Oncology, Gastroenterology, Hepatology, Pulmonology, and Infectious Diseases), University Cancer Center Leipzig (UCCL), University of Leipzig Medical Center, Leipzig, Germany; 3 Department of Visceral, Vascular, Thoracic and Transplant Surgery, University of Leipzig Medical Center, Leipzig, Germany; 4 Medical ICU, University of Leipzig Medical Center, Leipzig, Germany

**Keywords:** Cholangiocarcinoma, Thromboembolism, Risk prediction, ONKOTEV score, Khorana score

## Abstract

**Background:**

Patients with cancer are at increased risk of thromboembolic events contributing significantly to cancer-related morbidity and mortality. Because cholangiocarcinoma is a rare type of cancer, the incidence of thromboembolism in this patient population is not well defined.

**Methods:**

Patients with cholangiocarcinoma treated at the University Cancer Center Leipzig between January 2014 and December 2018 were analyzed retrospectively regarding the incidence of arterial and venous thromboembolism.

**Results:**

A total of 133 newly and consecutively diagnosed patients were included, of whom 22% had stage IV disease. Thromboembolism was diagnosed in 39 (29.3%), with 48% of the events occurring between 60 days prior and 30 days after the initial diagnosis. Arterial thrombosis accounted for 19% and portal venous thrombosis for 33% of the events, while the rest of events occurred in the non-portal venous system. In multivariable analysis, an ONKOTEV score ≥ 2 was the only independent predictor for thromboembolism. Serum CA 19-9 was available in 87 patients (65.4%). In this subgroup, CA 19-9 above the median of 97.7 U/ml and vascular or lymphatic compression were independent predictors for thromboembolism in the first year and CA 19-9 alone remained a significant predictor over the whole observation period. An ONKOTEV score ≥ 2 and increasing age were predictors of survival.

**Conclusions:**

A very high thromboembolic risk was observed in cholangiocarcinoma, comparable to the risk situation in pancreatic and gastric cancer. The ONKOTEV score and serum CA 19-9 are independent predictors of thromboembolic events. Prospective validation of our observations in this patient population is warranted.

**Supplementary Information:**

The online version contains supplementary material available at 10.1007/s00432-021-03794-1.

## Introduction

Venous and arterial thromboembolism (VTE, ATE) are frequent complications in patients with cancer. The incidence of VTE in patients with cancer is significantly higher than in the general population, with reported annual rates between 0.5 and 20%, depending on the specific cancer subpopulation, compared to annual incidence rates of 0.1–0.2% in non-cancer patients (Heit 2015; Horsted et al. 2012). VTE is also among the leading causes of death in cancer patients. The occurrence of thrombotic events is a negative prognostic factor beyond direct VTE-related mortality, underlining the complex interaction between the hemostatic system and malignancy (Chew et al. 2006; Khorana et al. 2007; Sorensen et al. 2000).

Several risk assessment models (RAM) for the stratification of VTE risk have been introduced. Most RAM build on the Khorana score (Khorana et al. 2008), which classifies gastric and pancreatic cancer as very high-risk tumor entities with thromboembolism (TE) rates of 15–30%, lymphoma or lung, gynecologic, bladder and testicular cancers as high risk and all other cancer types as low risk (Ay et al. 2010; Cella et al. 2017; Godinho et al. 2020; Khorana et al. 2008; Pabinger et al. 2018). In addition, high platelet count, low hemoglobin, leukocytosis, and a body mass index (BMI) > 35 kg/m^2^ are predictors for the calculation VTE risk in the Khorana score. The Protecht score added points for gemcitabine- or platin-based chemotherapy to the Khorana score (Verso et al. 2012). The ONKOTEV score included metastatic disease and vascular/lymphatic compression by the tumor as well as a history of VTE as predictors for VTE, which were equally weighted as a Khorana score > 2. The ONKOTEV score has proved to be highly predictive for VTE in patients with pancreatic cancer (Godinho et al. 2020).

Cholangiocarcinoma (CC) is a relatively rare type of cancer. Due to the low incidence, patients with CC were not included into the calculation of currently established RAMs. Thus, CC is counted as a low-risk entity for the prediction of VTE. Knowledge on the TE risk in patients with CC is limited to case reports, small case series (Blasi et al. 2018; Blum et al. 2016; Jang et al. 2006; Sasaki et al. 2020; Schorling et al. 2020; Yuri et al. 2014) and two retrospective case–control studies. Jeon et al. reported a VTE rate of 14.7% within a median follow-up of 14.4 months after initial diagnosis (Jeon et al. 2012), while 19.4% of patients undergoing hepatic resection had portal vein thrombosis in another study (Lu et al. 2016).

The aim of this study was to provide more evidence on the incidence and risk factors of VTE and ATE in patients with CC. In addition, the Khorana, Protecht (Verso et al. 2012) and ONKOTEV scores were evaluated regarding their performance in CC.

## Patients and methods

The prospectively maintained database of the University Cancer Center Leipzig (UCCL) was screened for patients with the diagnosis of CC treated between January 1st 2014 and December 31st 2018. Data regarding sex, age, medical history, tumour localisation, disease stage, treatment biomarkers including blood count, liver and renal function parameters at baseline as well as thromboembolic complications were analysed.

### Calculation of risk factors for thrombosis

Because thrombosis may precede the diagnosis of cancer (Navi et al. 2019a; White et al. 2005), all TE occurring within 60 days prior and all TE after the primary diagnosis of CC were considered cancer-associated. For the calculation of risk factors associated with tumor site or biomarkers, all thromboembolic events were taken into account. For the calculation of chemotherapy- or surgery-associated TE, only events within 6 months after the initiation of first or second-line chemotherapy and within 3 months after a surgical treatment were included and patients diagnosed with TE prior to therapy initiation were excluded. For the comparison between different treatment strategies, all thromboembolic events within 1 year after treatment initiation were included and patients diagnosed with TE prior to treatment were excluded. The calculation of the Khorana, Protecht and ONKOTEV scores is shown in the Supplementary Table 1.

### Statistics

Descriptive statistics are displayed as median with absolute range or interquartile range (IQR) for quantitative variables and numbers (percentages) for qualitative data. Intergroup comparisons were performed using either *t* test for normally distributed data or Mann–Whitney *U* test otherwise. Categorical variables are shown with frequency (%) and compared using Chi-square or Fisher’s exact test. For the subgroup-analysis of frequencies of VTE, portal vein thrombosis (PVT) and ATE, patients with thrombotic events other than the analyzed were excluded. *p* values < 0.05 are considered statistically significant. The Kaplan–Meier estimation was used for survival analysis and the distribution was compared with log rank test. The Kaplan–Meier estimation for the development of TE was calculated for the whole observation period and in a second step, the follow-up of each patient was set to 6 months after the initial diagnosis. The time point was chosen, because most of the recently published studies evaluating the primary prophylaxis with low molecular weight heparin and direct oral anticoagulants had a follow-up period of 6 months (Bosch et al. 2020; Carrier et al. 2019; Khorana et al. 2019). A multivariable analysis for the development of TE and survival was conducted using a Cox-regression model. Statistical analysis was performed using the software SPSS version 25 (SPSS Inc, Chicago, IL).

### Ethical considerations

The study was approved by the Ethics committee of the University of Leipzig (reference 180/20-ek), and conducted according to the declaration of Helsinki.

## Results

We identified 137 patients with CC consecutively diagnosed and treated between 01.01.2014 and 31.12.2018 at the Leipzig University Hospital. Four patients were excluded due to lack of histology (*n* = 1), concomitant pancreatic cancer (*n* = 1), concomitant hepatocellular carcinoma (*n* = 1) and a histology showing neuroendocrine cancer (*n* = 1). Finally, 133 patients were included into the analysis. Median follow-up was 8.6 (IQR 3.7–18.0) months for all patients and 11.0 (IQR 5.2–21.8) months for the 66 patients who were alive at last follow-up. Baseline characteristics are given in Table 1.

**Table 1:** Baseline characteristics and treatment of 133 patients with cholangiocarcinoma

Baseline characteristics			
Female gender	*n* (%)	61	45.9%
Age	Median (range)	67	31–82
BMI	Median (range)	25.5	16–43
Diagnosis			
Intrahepatic CC	*n* (%)	77	57.9%
Perihilar CC (Klatskin tumor)	*n* (%)	48	36.1%
Distal CC	*n* (%)	8	6.0%
Grading			
Grade I	*n* (%)	1	0.8%
Grade II	*n* (%)	53	39.8%
Grade III	*n* (%)	75	56.4%
Grading not available	*n* (%)	4	3.0%
Stage according to UICC			
I	*n* (%)	13	9.8%
II	*n* (%)	35	26.3%
III	*n* (%)	54	40.6%
IV	*n* (%)	30	22.6%
Staging not performed	*n* (%)	1	0.8%
Vascular/lymphatic compression	*n* (%)	34	25.6%
Khorana Score			
0	*n* (%)	72	54.1%
1	*n* (%)	49	36.8%
2	*n* (%)	8	6.0%
3	*n* (%)	4	3.0%
ONKOTEV score			
0	*n* (%)	52	39.1%
1	*n* (%)	60	45.1%
2	*n* (%)	20	15.0%
3	*n* (%)	1	0.8%
Treatment			
Surgery	*n* (%)	96	72.2%
Without tumor resection	*n* (%)	22	16.5%
With tumor resection	*n* (%)	74	55.6%
Chemotherapy	*n* (%)	50	37.6%
Alone	*n* (%)	8	6.0%
With surgery ± other therapies	*n* (%)	37	27.8%
With other therapies alone	*n* (%)	5	3.8%
Photon radiotherapy	*n* (%)	15	11.3%
SIRT/TACE/Chemosaturation	*n* (%)	27	20.3%
Photodynamic therapy	*n* (%)	4	3.0%
Best supportive care alone	*n* (%)	11	8.3%

*CC* cholangiocarcinoma, *SIRT* selective internal radiation therapy, *TACE* transarterial catheter chemoembolization, *ICC*: Union internationale contre le cancer

### Antithrombotic treatment at primary diagnosis

A total of 32 patients (24.1%) received antithrombotic treatment at the time of diagnosis. Most patients received acetyl salicylic acid (aspirin, ASA, *n* = 18) or a direct oral anticoagulant (DOAC, *n* = 9) mainly for atrial fibrillation and coronary artery disease. Detailed information on antithrombotic treatment at the time of diagnosis, indication and history of thromboembolic events are displayed in Table 2.

**Table 2:** Antithrombotic medication at initial diagnosis, indication and history of thrombotic events

Antithrombotic medication at time of initial diagnosis of cholangiocarcinoma	*n*	%
No	101	75.9
ASA	18	13.5
Direct oral anticoagulant	9	6.8
Low molecular weight heparin	1	0.8
Vitamin K antagonist	4	3.0
Indication for antithrombotic medication*		
Atrial fibrillation	12	9.0
Coronary heart disease	12	9.0
History of stroke	9	6.8
Peripheral artery disease	2	1.5
History of venous thromboembolism	2	1.5
Primary prophylaxis	2	1.5
History of thromboembolic events > 60 days prior to diagnosis		
Venous thromboembolism	7	5,3
Arterial thrombotic events	11	8.3
Stroke	7	5.3
Myocardial infarction	3	2.3
Recurrent stroke	1	0.8

*ASA* acetyl salicylic acid

*Seven patients had more than one indication for anticoagulation

A total of 39 of 133 patients (29.3%) had venous or arterial thromboembolic events, diagnosed within a median of 40 days after the primary diagnosis of CC (range 57 days prior to 980 after the diagnosis of CC). Three patients had two thromboembolic events. This was a myocardial infarction followed by VTE in one, stroke followed by VTE in another and portal vein thrombosis followed by VTE in a third patient. Another patient experienced a postoperative thrombosis of the portal vein combined with thrombosis of the hepatic artery and the coeliac trunk, which was counted as one thrombotic event. Table 3 gives an overview over all 42 thrombotic events in these 39 patients.

**Table 3:** Distribution of all thrombotic events

	All events, *n* (%)	< 60 days prior and ≤ 30 days after initial diagnosis, *n* (%)	> 30 days after initial diagnosis, *n* (%)
All events	42 (31.6)	20 (15.0)	22 (16.5)
Venous thrombotic events	33 (24.8)	15 (11.3)	18 (13.5)
DVT/PE	16 (12.0)	6 (4.5)	10 (7.5)
Portal vein thrombosis/VCI	14 (10.5)	8 (6.0)	6 (4.5)
Port-associated VTE	2 (1.5)	0 (0.0)	2 (1.5)
Phlebitis	1 (0.8)	1 (0.8)	0 (0.0)
Arterial thromboembolic events	8 (6.0)	5 (3.8)	3 (2.3)
Stroke	5 (3.8)	3 (2.3)	2 (1.5)
Peripheral arterial thrombosis	1 (0.8)	0 (0.0)	1 (0.8)
Hepatic artery thrombosis	1 (0.8)	1 (0.8)	0 (0.0)
Myocardial infarction	1 (0.8)	1 (0.8)	0 (0.0)
Combined events			
Thrombosis of the portal vein, hepatic artery and coeliac trunk	1 (0.8)	0 (0.0)	1 (0.8)

*DVT* deep vein thrombosis, *PE* pulmonary embolism, *VCI* vena cava inferior

**Fig. 1:**
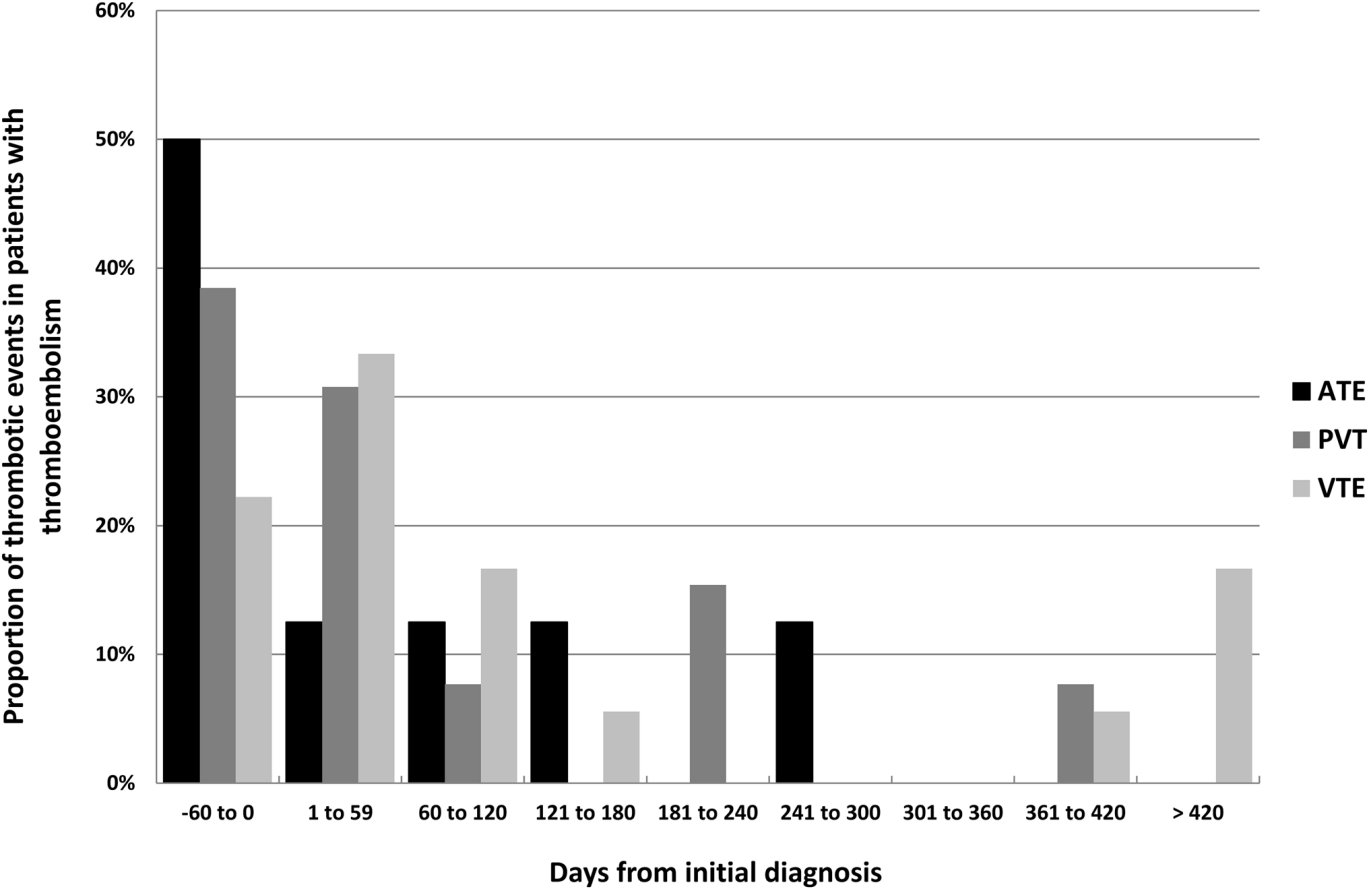
Distribution of thromboembolic events over time. One combined thrombotic event was ommited. *ATE* arterial thrombotic event, *PVT* portal vein thrombosis, *VTE* venous thromboembolism

The distribution of the thromboembolic events over time is shown in Fig. 1. The four events occurring later than 1 year after the primary diagnosis were all associated with persistent or progressive disease.

### Predictors of thromboembolic events

#### Biomarkers

Patients with C-reactive protein (CRP) above the normal range had a VTE rate of 33.0% compared to 17.9% in patients with a normal CRP at initial diagnosis, *p* = 0.081. Platelet count ≥ 350,000/µl, leucocyte count > 11,000/µl, and elevated bilirubin at primary diagnosis were not associated with an increased TE rate. CA 19-9 and CEA at primary diagnosis were measured in 87 and 66 patients, respectively. CA 19-9 was significantly higher in patients with a TE within the first year compared to those without TE [median: 324 (IQR 61–1567) U/ml vs. 83.3 (IQR 26–173) U/ml, *p* = 0.040], resulting in a 2.7-fold increased risk for TE within the first year in patients with a CA 19-9 above the median of 97.7 U/ml (Supplementary Fig. 2). For the whole observation period, CA 19-9 above the median was associated with a trend towards a higher rate of TE (22.7 vs. 41.9%, OR 3.6, *p* = 0.056). CA 19-9 was above the median in all five patients with ATE, in 6/14 (42.9%) patients with VTE and in 7/9 (77.9%) patients with PVT and known CA19-9 at initial diagnosis (*p* = 0.045 for the comparison between VTE and ATE). There was a higher median CA19-9 in patients with PVT (312 [IQR 146–1929] U/ml vs. 83 [IQR 29–282] U/ml, *p* = 0.043) and a trend towards a higher median CA19-9 in patients with ATE (409 [IQR 279–871] U/ml vs. 83 [IQR 29–282] U/ml, *p* = 0.065) compared to patients without TE, respectively. CEA was not predictive for TE.

#### Treatment

The risk of developing TE within 12 months after initiation of any cancer-related therapy was 19.5% (22/113 eligible patients), while the 6-month risk after initiation of chemotherapy was 22.7% (10/44 eligible patients, 6 VTE, 3 PVT, 1 ATE). The 3-month risk for postoperative TE was 12.2% (11/90 eligible patients, 6 VTE, 2 PVT, 2 ATE, 1 combined event) and 13.9% (10/62 eligible patients) after exclusion of patients without tumor resection. No patient who received best supportive care had TE after but 3/8 patients prior to the initial diagnosis of CC.

No difference in the 12-month risk of TE was seen between patients receiving chemotherapy versus no chemotherapy (22.7% vs. 17.4%; *p* = 0.627), surgical treatment (operation 20.9%, no operation 13.6%, *p* = 0.559) or a combination (chemotherapy alone 20.8%, chemotherapy and surgery 14.3%, *p* = 0.740). Out of 44 patients without TE prior to the initiation of chemotherapy, 7 patients received capecitabin or gemcitabine alone and 37 patients received a platinum derivative. The 6-month rate of TE was 28.6% in the capecitabin or gemcitabine group versus 21.6% in the platinum group (*p* = 0.649).

#### Cancer-associated risk factors

No significant difference in the rates of TE was seen between metastatic and localized disease (39.4% vs. 25.3%, *p* = 0.120), vascular or lymphatic compression (38.2% vs. 25.3%, *p* = 0.148), high-grade versus low and intermediate grade (29.3% vs. 25.9 *p* = 0.670) and extrahepatic versus intrahepatic CC (25.0% vs. 31.2%, *p* = 0.437). After exclusion of patients with ATE, 11/32 (34.4%) patients with vascular or lymphatic compression had VTE or PVT compared to 18/92 (19.6%) patients without vascular or lymphatic compression, *p* = 0.088, which was mainly due to a trend to a higher rate of PVT in these patients (22.2% vs. 8.6%, OR 3.02, *p* = 0.060).

Thromboembolic events in different patient groups are summarized in the Supplementary Table 2.

### TE rates according to Khorana, ONKOTEV and Protecht score

When CC was counted as low-risk cancer, 5/12 (41.7%) patients with Khorana score ≥ 2 had a thromboembolic event compared to 33/121 (27.3%) patients with Khorana score < 2, *p* = 0.292. There was no significant difference when CC was counted as high or very high-risk cancer entity. Khorana score was not predictive for VTE, PVT or ATE. When CC was counted as a low-risk cancer, patients with an ONKOTEV score ≥ 2 had a higher TE rate (9/18, 50%) compared to patients with a score < 2 (29/115, 25.2%), *p* = 0.030. The difference was still significant when CC was counted as high risk (10/21 vs. 28/112, *p* = 0.035). There was still a trend when CC was defined as a very high-risk entity (17/43 vs. 21/90, *p* = 0.053). An ONKOTEV score ≥ 2 was associated with a higher rate of PVT (35.7% vs. 8.5%, OR 5.97, *p* = 0.004) and a trend to a higher rate of ATE (25.0% vs. 6.5%, OR 4.78, *p* = 0.067) but not with a higher incidence of VTE (10.0% vs. 14.9%, OR 0.64, *p* = 1.00). Patients receiving chemotherapy with a Protecht score ≥ 3 had a TE rate of 47.8% compared to 29.6% in patients with a Protecht score < 3, *p* = 0.186. The rate of VTE, PVT and ATE was not different in the high and low-risk groups according to the Protecht score (Supplementary Table 2).

### Prediction of thrombotic events at different time points

**Fig. 2:**
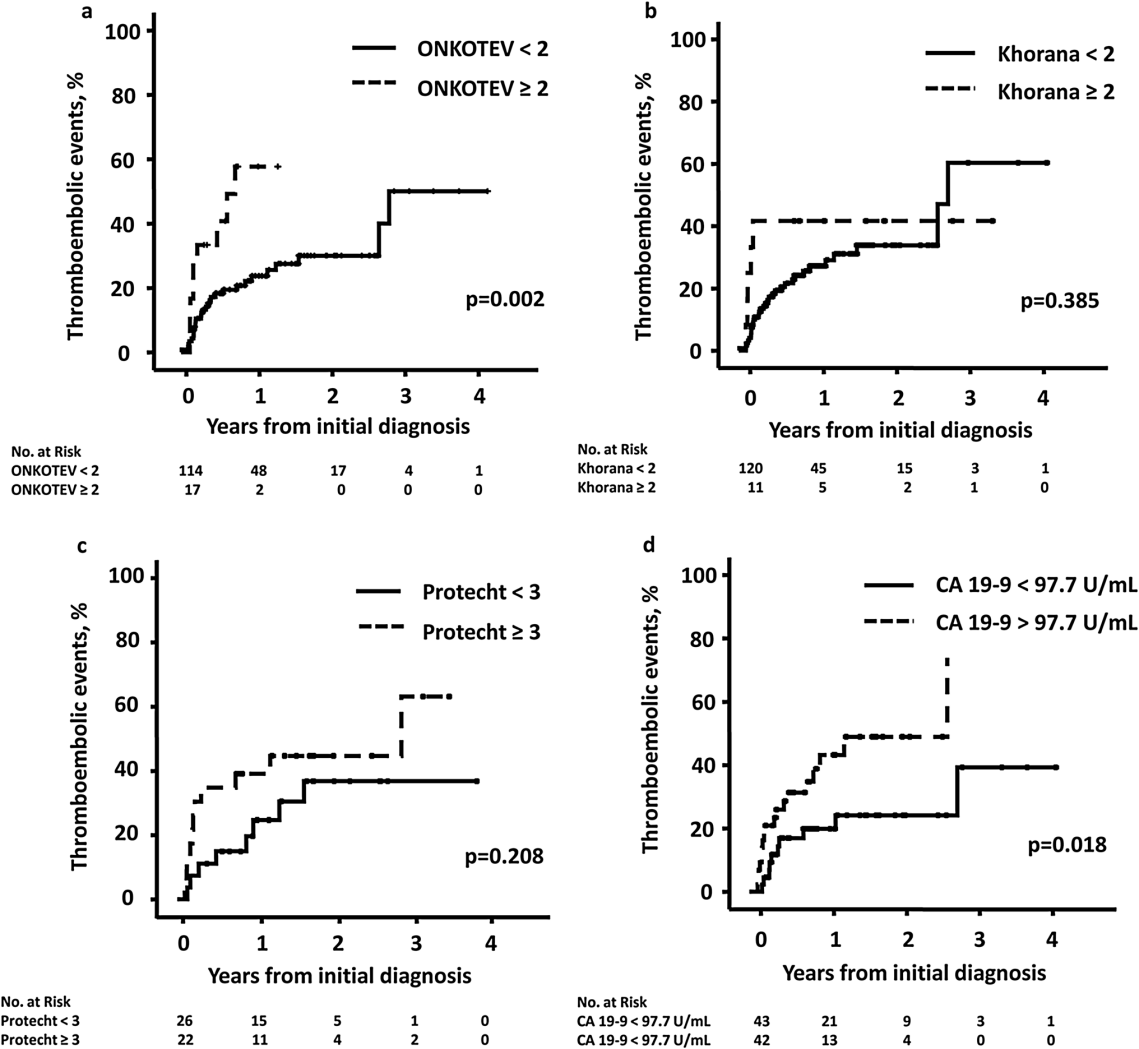
Thromboembolic events according to **a** ONKOTEV score, **b** Khorana score, **c** Protecht score, and **d** median CA 19-9 (97.7 U/mL)

Taking into account the entire observation period, the risk of developing TE at 1 year was 25.6% in patients with an ONKOTEV score < 2 and 57.7% in patients with an ONKOTEV score ≥ 2, *p* = 0.002 (Fig. 2a). This difference remained significant when CC was counted as high or very high-risk cancer entity (Supplementary Fig. 1). The risk of developing TE at 1 year in patients with a Khorana score < 2 was 27.2% compared to 41.7% in patients with a Khorana Score ≥ 2, *p* = 0.385 (Fig. 2b). The risk of developing TE at one and 2 years in patients receiving chemotherapy and a Protecht score < 3 was 24.7 and 36.8% compared to 39.1 and 44.7% in patients with a Protecht score ≥ 3, *p* = 0.208, respectively (Fig. 2c). The one- and two-year TE rate in patients with CA 19-9 above the median compared to those with CA 19-9 below the median was 43.2% vs. 20.0% and 48.9% vs. 24.1%, *p* = 0.018, respectively (Fig. 2d).

When the final follow-up of all patients for the Kaplan Meier estimation was set to 6 months after initial diagnosis, the TE rate was 19.1% for ONKOTEV < 2 vs. 44.4% for ONKOTEV ≥ 2 (*p* = 0.008), 20.9% for Khorana score < 2 vs. 41.7% for Khorana score ≥ 2 (*p* = 0.057), and 14.8% for Protecht < 3 vs. 34.8% for Protecht ≥ 3. (Supplementary Fig. 3). There was no difference after exclusion of arterial thromboembolic events.

### Survival

**Fig. 3:**
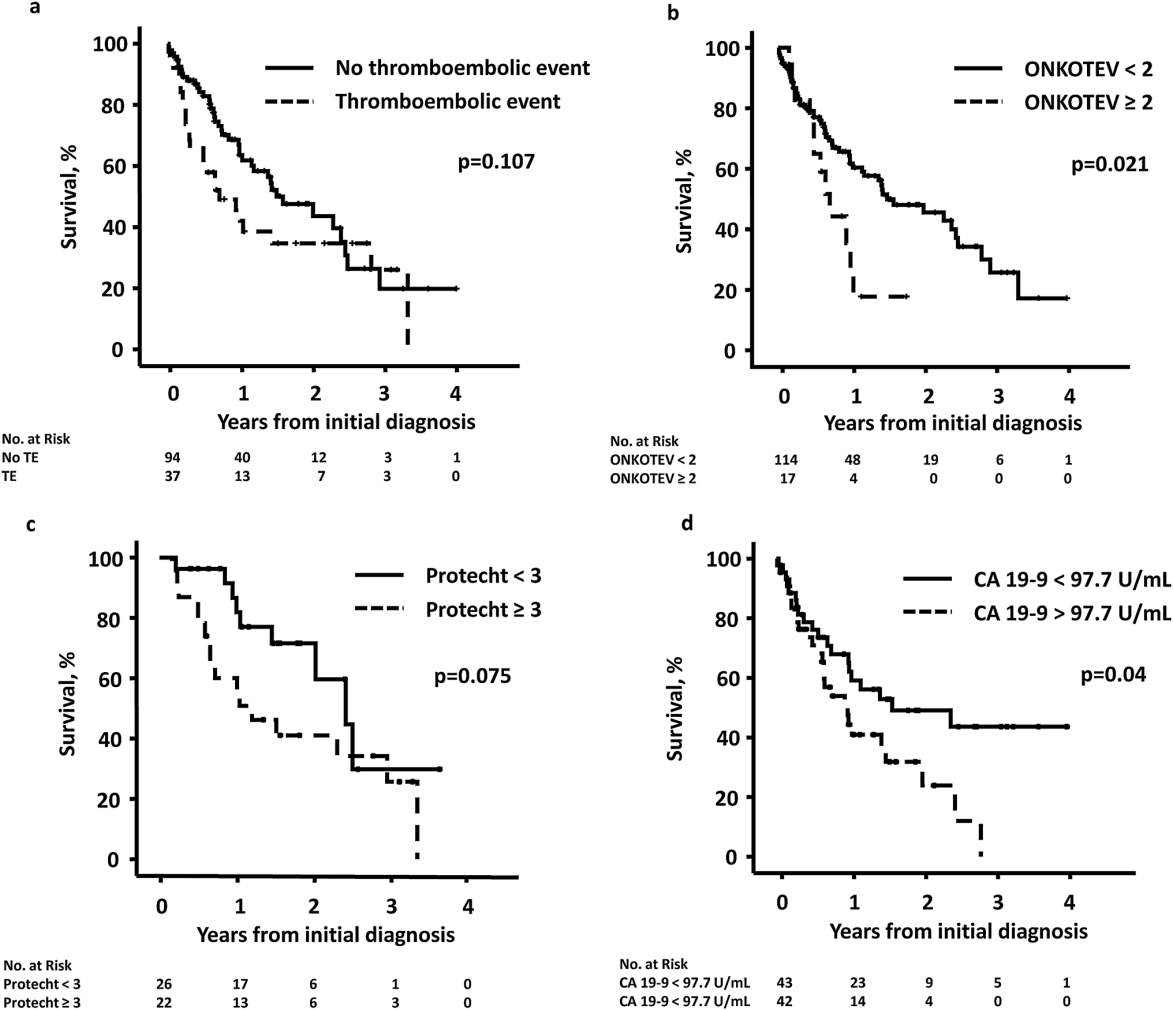
Survival according to **a** thromboembolic events, **b** ONKOTEV score, **c** Protecht score, and **d** median CA 19-9 (97.7 U/mL). *TE* thromboembolic event

At last follow-up, 34.2% with TE compared to 55.8% in patients without TE were alive, *p* = 0.034, odds ratio 2.4. Patients with surgical treatment had a median overall survival (OS) of 0.65 (95% CI 0.48–0.82) years vs. 2.5 [95% CI 1.9–3.1) years, *p* < 0.001. Median OS was not different in patients who underwent surgery with cancer resection in curative intention (3.0 [95% CI 0.86–5.07] years) or in patients who underwent exploratory laparotomy but were not resectable (2.5 [95% CI 0.90–4.12] years), p = 0.859. The median OS for patients with TE was 0.7 [95% CI 0.3–1.2] years vs. 1.6 [95% CI 1.0–2.2] years for those without TE, *p* = 0.107. Survival rates at 1 year and 2 years were 45.5% and 34.7%, respectively, for patients with TE compared to 63.6% and 47.5% for those without a TE (Fig. 3a). Patients with a low ONKOTEV score had a significantly better survival. The 1-year overall survival in patients with an ONKOTEV score < 2 vs. ≥ 2 for CC counted as low-risk tumor was 64.3% vs. 35.4%, *p* = 0.021 (Fig. 3b). This difference was still significant, when CC was counted as a high or very high-risk tumor: 64.3% vs 39.5%, *p* = 0.04 and 67.6% vs. 38.3%, *p* = 0.001, respectively. The median OS in patients receiving chemotherapy and a Protecht score ≥ 3 was 1.2 [95% CI 0.46–1.9] years compared to 2.4 [95% CI 1.6 vs. 3.3] years in patients with a Protecht score < 3, *p* = 0.075 (Fig. 3c). The 1- and 2-year survival in patients with CA 19-9 below the median compared to patients with CA 19-9 above the median was 65.0% vs. 44.4% and 49.0% vs. 31.8%, *p* = 0.040, Fig. 3d. There was no difference in survival of patients with a low or high Khorana score, regardless of the risk status. The median OS in different patient groups is summarized in the Supplementary Table 3.

### Predictors for poor survival in patients with thrombotic events

Survival was less than 1 year in 25 and more than 1 year in 13 patients with TE. Three patients with TE surviving less than 1 year were treated with best supportive care, the remaining patients received at least one treatment strategy. Patients with TE who survived less than 1 year were older (median age: 72 [IQR: 65–75] years vs. 64 [IQR 55–71] years), *p* = 0.048, had a lower median BMI (24 [IQR: 21–28] kg/m^2^ vs. 26 [IQR: 24–31] kg/m^2^, *p* = 0.023) and received chemotherapy less frequently (36.0% vs. 76.9%, *p* = 0.038). Survival was less than 1 year in 50% of the patients with VTE, 62% of the patients with PVT and 100% of the patients with ATE. No other significant risk factors for poor prognosis in patients with TE were found.

### Multivariable analysis

A multivariable Cox-regression model was performed for the prediction of thromboembolic events including the potential cancer-associated risk factors: intrahepatic vs. extrahepatic CC, stage IV disease, vascular or lymphatic compression, Khorana score > 2 and ONKOTEV score ≥ 2, the blood biomarkers CRP > upper limit of normal (ULN) and serum bilirubin > ULN and the baseline risk factors: age and BMI as continuous variables, history of TE and pre-existing anticoagulation with VKA or DOAC. For the prediction of survival, TE events were added. ONKOTEV score ≥ 2 was the only independent predictor for TE in the first year and in the whole observation period. ONKOTEV score ≥ 2 and age (increased relative risk of 3.4 [95% CI 0.6–6.2%] % per year) were independent predictors for survival. When only the 87 patients with known CA19-9 values were included into the analysis, CA 19-9 above the median and vascular or lymphatic compression were independent predictors for TE in the first year, while CA19-9 above the median for TE in the whole observation period and ONKOTEV score ≥ 2 and age for OS. The results of the multivariable analysis are summarized in Table 4.

**Table 4:** Results of the multivariable Cox-regression analysis

	TE in the first year			TE in the whole observation period			Survival		
	Predictor	HR (95% CI)	*p*	Predictor	HR (95% CI)	*p*	Predictor	HR (95% CI)	*p*
All patients	ONKOTEV ≥ 2	3.1 (1.4–6.7)	0.004	ONKOTEV ≥ 2	3.0 (1.4–6.5)	0.005	ONKOTEV ≥ 2	1.9 (1.0–3.7)	0.048
							Age (per year)	1.034 (1.006–1.062)	0.031
Patients with known CA 19-9	CA 19-9 > median	2.4 (1.0–5.7)	0.043	CA 19-9 > median	2.5 (1.1–5.7)	0.025	ONKOTEV ≥ 2	2.2 (1.1–4.7)	0.037
	Vascular or lymphatic compression	2.3 (1.0–5.3)	0.048				Age (per year)	1.031 (1.002–1.062)	0.039

The multivariable analysis included: age (continuous variable) and BMI (continuous variable), history of TE, pre-existing anticoagulation with VKA or DOAC, intrahepatic vs. extrahepatic disease, stage IV disease, vascular or lymphatic compression, Khorana score > 2 and ONKOTEV score ≥ 2, CRP > ULN, serum bilirubin > ULN

*TE* thromboembolism, *BMI* body mass index, *VKA* vitamin K antagonist, *DOAC* direct oral anticoagulant, *CRP* C-reactive protein, *ULN* upper limit of normal

## Discussion

This study on consecutive patients treated at a tertiary referral cancer center in Germany revealed a TE rate in patients with CC of 29.3%. Approximately 50% of the thromboembolic events occurred between 2 months prior and one month after the initial diagnosis, arterial events accounted for approximately 20% of all thromboembolic events and almost 42% of all VTEs occurred in the portal vein. The largest retrospective study to date on patients with CC reported 40 (14.7%) TEs in 273 patients (Jeon et al. 2012). In that study, only 10 patients (25%) had TE at initial diagnosis and only 14 (35%) had VTE. The remaining cases were PVT (*n* = 18, 45%) and thrombosis of the inferior vena cava (*n* = 4, 10%) and the hepatic veins (*n* = 4, 10%). However, there were no ATEs reported. Two other studies focused on PVT in patients undergoing liver resection for CC. Lu and colleagues retrospectively analyzed data from 303 patients at the time of liver resection and found tumor associated PVT in 19.3%. Patients with a PVT had poorer survival and were more likely to have lymph node metastasis and elevated bilirubin levels (Lu et al. 2016). In a prospective case series of 27 CC patients admitted for liver resection without history of thrombosis, 6 (22.2%) patients had TE (2 VTE and 4 PVT) within 60 days after surgery (Blasi et al. 2018). In our cohort, TE occurred in only 13.9% of all patients after tumor resection and these were mainly non-portal VTE.

### Arterial thromboembolic events

The true incidence of ATE in CC is unknown. We found ATEs in 6.0% of our patients. A few case reports described CC patients having ATE (Dunn et al. 2017; Sasaki et al. 2020; Yuri et al. 2014). In our study, 50% of ATE preceded the diagnosis of CC. A large study on 374,331 patients ≥ 67 years of age diagnosed with different types of cancer showed that the ATE rate within 6 months prior to the diagnosis of cancer is progressively increasing, with a sixfold increased risk compared to an age-matched cohort within the last month before cancer diagnosis (Navi et al. 2019a). Other studies showed a 2.2 fold increased risk of cancer diagnosis within 6 months after myocardial infarction (Rinde et al. 2017) and a 3.3 fold increase within 6 months after lower limb arterial thrombosis (Sundbøll et al. 2018). The risk of ATE is still elevated after cancer diagnosis but the highest risk is within the first month after cancer diagnosis (Navi et al. 2017, 2019b). Data from a US registry of patients with incident cancer showed a cumulative 6-month incidence of myocardial infarction of 2.6–3.1% and of ischemic stroke of 3.5–3.8% in patients with gastric, pancreatic and colorectal cancer (Navi et al. 2017). In our cohort, 4 (3.0%) ATE occurred prior or at the time of initial diagnosis, and 3 (2.3%) ATE occurred within 6 months after the diagnosis of CC. A direct comparison between the results is not feasible due to the comparably low number of patients included in our study and the rather short observation period. Nevertheless, our data indicate that the risk of ATE in CC patients seems to follow the same pattern known from other tumor entities, with the highest risk shortly before and after the initial diagnosis.

### Prediction of thromboembolic events

ONKOTEV score was the only independent predictor for TE in the first year of the observation period. The Khorana and the Protecht score showed a trend to a higher rate of TE within the first 6 months after initial diagnosis in the Kaplan Meier estimation. The data regarding the Protecht score should be interpreted with caution due to the limited number of 50 patients receiving chemotherapy, since the Protecht score can be assessed in that patient cohort only. In addition, only the Protecht score but not the ONKOTEV and the Khorana score are validated for the prediction of both arterial and venous thrombotic events. We found that an ONKOTEV score ≥ 2 was associated with a trend to a higher ATE rate and that the TE rates were not different after exclusion of ATE. Lymphatic or vascular compression was associated with a trend to a higher rate of PVT and was an independent predictor for TE in the first year in the subgroup of patients with known CA 19-9. The fact that lymphatic or vascular compression is part of the ONKOTEV score might be the reason why the ONKOTEV score performed better in our study than the Khorana score.

Due to the lack of data regarding the performance of RAM for the prediction of TE in CC patients, the interpretation might be extrapolated from other tumor entities. Although patients with pancreatic and gastric cancer are better predicted by the Khorana score than lung cancer patients (van Es et al. 2020), the ONKOTEV score is highly predictive for VTE in patients with pancreatic cancer (Godinho et al. 2020). In that study, a Khorana score > 2 did not predict TE while the ONKOTEV score and its components vascular compression and metastatic disease did. Similar to our study, 51% of patients had abdominal thrombosis that was mainly caused by vascular compression.

Apart from the ONKOTEV score, we have found a trend towards a positive association between TE rate and elevated plasma CRP levels. The association of CRP with TE in CC was already described in a Korean cohort of patients (Jeon et al. 2012). The strongest known predictive biomarker for TE is d-dimer. A recently published RAM included only d-dimer and the tumor entity to predict a 6-month VTE rate in cancer patients (Pabinger et al. 2018). Unfortunately, due to the retrospective design of our study, no d-dimer levels could be assessed, as this parameter is not part of the routine laboratory in our center. However, as there is a well-known correlation between d-dimer and CRP, patients with elevated CRP are likely to have also elevated d-dimer levels. The value of d-dimer in the prediction of TE in patients with CC should be addressed in prospective studies.

CA 19-9 was associated with an increased risk of TE in our study and it was also a predictor of poor survival in the univariate analysis. CA-19-9 is a well-established prognostic factor in patients with CC (Ali et al. 2007; Bergquist et al. 2016; Hahn et al. 2020; Li et al. 2017; Wang et al. 2013), but there are to the best of our knowledge no reports describing an association between CA 19-9 and the risk of TE in CC patients. It has been shown elsewhere that CA 19-9 correlated with the severity of VTE in patients with pancreatic cancer (Woei-A-Jin et al. 2016). CA 19-9 may serve as a surrogate marker to quantify mucins in blood as it binds to apomucins (Yue et al. 2011). Lu and colleagues showed that patients with mucinous CC have significantly higher CA 19-9 levels, were more likely to have vascular invasion and had poorer overall and disease free survival after hepatic resection (Lu et al. 2019). The injection of purified mucins into mice resulted in the formation of tissue factor independent platelet rich microthrombi via P-selectin and L-selectin activation (Wahrenbrock et al. 2003). The fact that CA 19-9 was above the median in all patients with ATE, but only in 42.9% of VTE patients, supports the assumption of a mainly platelet-dependent emergence of TE in patients with elevated CA 19-9 that might be related to a mucinous subtype.

### Comparison with other cancer types

VTE rates in patients with pancreatic cancer ranged between 10% within 3 months after initiation of chemotherapy in a prospective study (Pelzer et al. 2015) and 20–40% in different retrospective cohort studies (Epstein et al. 2012; Godinho et al. 2020; Khorana et al. 2013; Kruger et al. 2017; Menapace et al. 2011). The incidence of VTE in patients with gastric (GC) and esophageal cancer (EC) ranges between 9 and 20% (Aonuma et al. 2019; Fuentes et al. 2018; Khorana et al. 2013; Lyman et al. 2013; Marshall-Webb et al. 2017; Starling et al. 2009). In contrast, the incidence of VTE in patients with colorectal cancer (CRC) is 10–12% in North-American cohorts (Khorana et al. 2013; Lyman et al. 2013), while it is 17% in an Asian cohort (Aonuma et al. 2019). The rate of TE was almost 30% in our study population and seems to be in the range of pancreatic and gastric cancer. We, therefore, propose that CC should be counted as a high-risk tumor entity. However, this should be validated in independent cohorts.

### Survival

Survival in our cohort was predicted by a high ONKOTEV score and age, while TE failed to be predictive for survival in the Kaplan–Meier estimation. TE is a well-established unfavorable prognostic factor for survival in cancer patients and was associated with poor survival in CC patients (Jeon et al. 2012). The reason why TE was not associated with poor survival in our cohort might be explained by the limited number of patients included. However, this highlights the predictive value of the ONKOTEV score which was an independent predictive factor even after the inclusion of patients with known CA 19-9 into the analysis. As mentioned above, the inclusion of lymphovascular invasion in the ONKOTEV score may be the reason why the ONKOTEV score is highly predictive in patients with CC, because lymphovascular invasion was shown to be an adverse prognostic factor for overall and disease free survival in patients with hilar CC type Bismuth IV (Li et al. 2017).

### Limitations

Our study has several limitations due to its retrospective design, conduct in a monocentric setting, and the limited number of patients included into the analysis. Thus, the influence of minor risk factors may have been missed. In addition, CA 19-9 was only available in 65% of patients, which limits conclusions from the multivariate analysis. Moreover, D-dimer as the strongest predictive blood-based biomarker for TE in cancer was not available in our study. Therefore, we were unable to calculate the d-dimer-based CATS score (Pabinger et al. 2018). In addition, other RAMs like the CONKO score (Pelzer et al. 2015) could not be calculated because the WHO performance score was not documented in our study and the Protecht score could only be calculated for a limited number of patients receiving chemotherapy. Nevertheless, and to the best of our knowledge, this study represents the first distinct and largest analysis of thromboembolic incidence and risk factors in patients with newly diagnosed CC.

## Conclusion

Our study showed that patients with CC have a high to very high risk of TE, comparable to that of patients with pancreatic and gastric cancers. In contrast to most other studies in cancer patients focusing mainly on venous TE, ATE accounted for about 20% of the TE in our cohort. In addition, half of the TE occurred between 2 months prior and one month after the initial diagnosis. An ONKOTEV score ≥ 2 and CA 19-9 above the median are independent predictors for TE, while CA 19-9 was particularly predictive for ATE. The good performance of the ONKOTEV score in CC might be explained by the high rate of PVT in CC (42% of all venous thrombotic events) most likely caused by lymphatic and vascular compression.

Based on our findings, primary prophylaxis should be considered in patients with CC but particularly in those with high CA 19-9 levels and a high ONKOTEV score. Prospective studies including d-dimer and more RAMs are warranted in patients with CC to confirm these data.

## Supplementary Information

Below is the link to the electronic supplementary material.

Supplementary file1 (PDF 287 KB)

## Data Availability

Please contact author for data requests.
