# Supplementary material for: Predictors for thromboembolism in patients with cholangiocarcinoma
Source: J Cancer Res Clin Oncol. 2021 Sep 9;148(9):2415–26. doi: 10.1007/s00432-021-03794-1 (PMC9349130; doi:10.1007/s00432-021-03794-1)
Supplement: Supplementary file 1 — Supplementary file1 (PDF 287 KB) [file 432_2021_3794_MOESM1_ESM.pdf]

Predictors for thromboembolism in patients with cholangiocarcinoma

Christian Pfrepper[1,] Maren Knödler[2], Ruth Maria Schorling[2], Daniel Seehofer[3], Sirak Petros[1,4], Florian Lordick[2]

[1] Division of Hemostaseology, Department of Hematology, Cellular Therapy and Hemostaseology, University of Leipzig Medical Center, Leipzig, Germany

[2] Department of Medicine (Oncology, Gastroenterology, Hepatology, Pulmonology, and Infectious Diseases), University Cancer Center Leipzig (UCCL), University of Leipzig Medical Center, Leipzig, Germany

[3] Department of Visceral, Vascular, Thoracic and Transplant Surgery, University of Leipzig Medical Center, Leipzig, Germany

[4] Medical ICU, University of Leipzig Medical Center, Leipzig, Germany

Corresponding author:

Dr. Christian Pfrepper

Division of Hemostaseology

Department of Hematology, Cellular Therapy and Hemostaseology

University of Leipzig Medical Center, Leipzig, Germany

Liebigstr. 20, 04103 Leipzig, Germany

Phone: +49 341 9712700, Fax: +49 341 9712709

E-Mail: christian.pfrepper@medizin.uni-leipzig.de

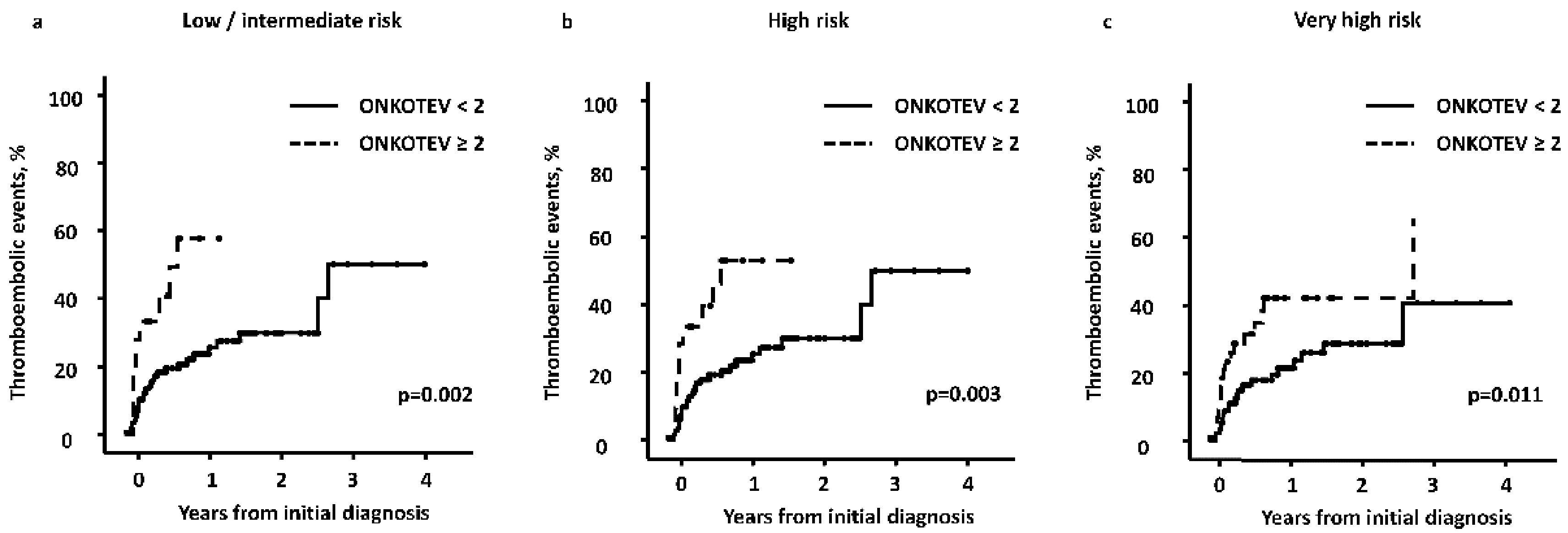

Supplementary figure 1: Thromboembolic events according to ONKOTEV score when cholangiocarcinoma was counted as a) low or intermediate risk, b) high risk, and c) very high risk

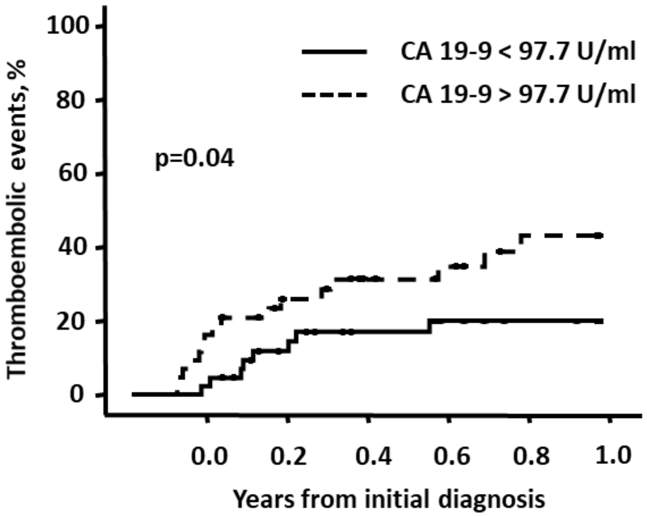

Supplementary figure 2: Thromboembolic events according to CA 19-9 for the first year after diagnosis

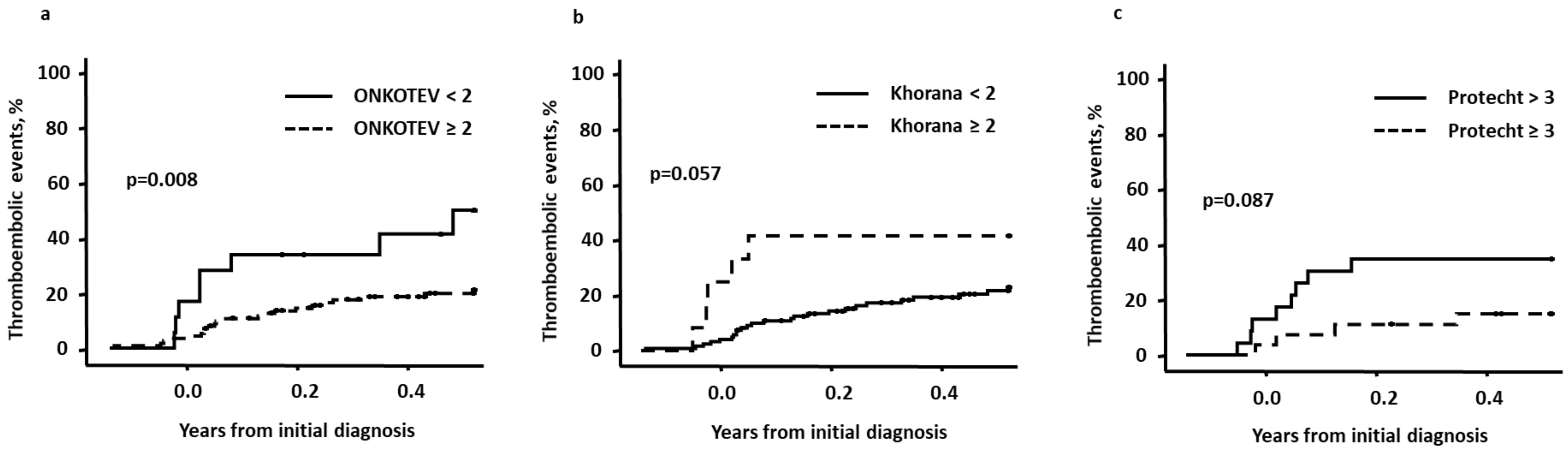

Supplementary figure 3: Thromboembolic events for the first 6 months after diagnosis according to a) ONKOTEV score, b) Khorana score, and c) Protecht score

Supplementary table 1: Calculation of risk assessment models

Score	Predictor	Points
**Khorana-score**	Very high risk cancer (stomach, pancreas)	2
	High risk cancer (lung, lymphoma, gynecologic, bladder, testicular)	1
	Prechemotherapy platelet count ≥ 350 Gpt/L	1
	Hemoglobin level < 10 g/dL or use of red cell growth factors	1
	Prechemotherapy leukocyte count > 11 Gpt/L	1
	BMI ≥ 35 kg/m^2	1
**PROTECHT-score**	Khorana Score	0-6
	cisplatin or carboplatin or gemcitabine chemotherapy	1
	combination of cisplatin and / or carboplatin and / or gemcitabine chemotherapy	2
**ONKOTEV-score**	Khorana Score > 2	1
	Previous venous thromboembolism	1
	Metastatic disease	1
	Vascular / lymphatic macroscopic compression	1

Supplementary table 2: Thrombotic events in different patient groups; * cholangiocarcinoma was counted as low risk, # patients without chemotherapy received 0 points for chemotherapy, n. a.: not applicable, OR: odds ratio, CI: confidence interval, ULN: upper limit of normal

		**All thrombotic events**				**Venous thromboembolism**				**Portal vein thrombosis**				**Arterial thrombotic events**			
**Predictor**	**Variable**	**Event rate, %**	**OR**	**95% CI**	**p**	**Event rate, %**	**OR**	**95% CI**	**p**	**Event rate, %**	**OR**	**95% CI**	**p**	**Event rate %**	**OR**	**95% CI**	**p**
**Patients**																	
Age	> 65 years vs. < 65 years	32.5 vs. 22.6	1.7	0.74-3.64	0.218	14.3 vs. 14.6	1.0	0.34-2.84	0.965	12.9 vs. 10.9	1.2	0.37-4.00	1.000	0.0 vs. 14.3	n. a.		*0.011*
BMI	> 30 kg/m2 vs < 30 kg/m2	23.8 vs. 30.8	0.7	0.30-1.63	0.409	13.5 vs. 14.9	0.9	0,29-2.80	0.848	11.1 vs. 12.5	0.9	0.25-3.06	1.000	3.0 vs. 11.3	0.3	0.03-2.05	0.266
**Biomarkers**																	
CA 19-9	> median vs. < median	41.9 vs. 22.7	2.5	0.96-6.20	*0.056*	19.4 vs. 19.0	1.0	0.31-3.31	0.974	21.9 vs. 5.6	4.8	0.91-24.9	*0.073*	0.0 vs. 16.7	n. a.		*0.019*
CRP	> ULN vs. normal range	33.0 vs. 17.9	2.3	0.89-5.67	*0.081*	14.9 vs. 13.5	1.1	0.36-3.49	1.000	16.0 vs. 3.0	6.1	0.76-49.0	0.104	11.3 vs. 3.0	4.1	0.49-33.9	0.266
Bilirubin	> ULN vs. normal range	21.7 vs. 34.2	0.5	0.24-1.16	0.110	6.0 vs. 21.3	0.2	0.06-0.88	*0.029*	7.8 vs. 15.8	0.5	0.13-1.58	0.247	11.3 vs. 5.9	2.0	0.48-8.65	0.489
**Tumor**																	
Localization	extrahepatic vs. intrahepatic	25.0 vs. 31.2	0.7	0.34-1.60	0.437	10.6 vs. 17.2	0.6	0.19-1.78	0.418	8.7 vs. 14.5	0.6	0.16-1.95	0.551	10.6 vs. 7.0	1.6	0.40-6.24	0.728
Grading	high grade vs. low grade	29.3 vs. 25.9	1.2	0.54-2.60	0.696	14.5 vs. 13.0	1.1	0.37-3.44	0.827	13.1 vs. 9.1	1.5	0.43-5.37	0.523	8.6 vs. 9.1	0.9	0.24-3.74	1.000
Metastasis	present vs. absent	39.4 vs. 25.3	1.9	0.84-4.42	0.120	20.0 vs. 12.9	1.7	0.52-5.40	0.355	20.0 vs. 9.8	2.3	0.68-7.85	0.170	13.0 vs. 7.5	1.9	0.43-8.06	0.414
Stage	III/IV vs. I/II	28.6 vs. 29.2	1.0	0.44-2.12	0.942	13.0 vs. 17.1	0.7	0.25-2.13	0.562	14.3 vs. 8.1	1.9	0.49-7.34	0.536	7.7 vs. 10.5	0.7	0.18-2.82	0.722
Stage	IV vs. I/II/III	40.0 vs. 25.5	2.0	0.83-4.58	0.123	21.7 vs. 12.6	1.9	0.59-6.22	0.319	18.2 vs. 10.6	1.9	0.52-6.78	0.462	14.3 vs. 7.3	2.1	0.48-9.26	0.384
vascular compression	present vs. absent	38.2 vs. 25.3	1.8	0.80-4.19	0.148	19.2 vs. 12.9	1.6	0.50-5.12	0.523	22.2 vs. 8.6	3.0	0.92-9.96	*0.060*	8.7 vs. 8.6	1.0	0.19-5.21	1.000
**Risk assessment models**																	
Khorana score	≥ 2 vs. 0-1	41.7 vs. 27.3	1.9	0.57-6.42	0.292	12.5 vs. 14.6	0.8	0.10-7.31	1.000	22.2 vs. 11.1	2.3	0.42-12.4	0.296	22.2 vs. 7.4	3.6	0.62-20.7	0.174
ONKOTEV score	≥ 2 vs. 0-1	50.0 vs. 25.2	3.0	1.07-8.19	*0.030*	10.0 vs. 14.9	0.6	0.08-5.40	1.000	35.7 vs. 8.5	6.0	1.61-22.2	*0.004*	25.0 vs. 6.5	4.8	1.02-22.4	*0.067*
Protecht Score	≥ 3 vs. 0-2 (all patients #)	45.8 vs. 24.8	2.6	1.03-6.40	*0.039*	23.5 vs. 12.8	2.1	0.59-7.52	0.265	27.8 vs. 8.9	3.9	1.12-13.9	*0.025*	13.3 vs. 7.9	1.8	0.34-9.64	0.614
Protecht score	≥ 3 vs. 0-2 (patients with chemotherapy)	47.8 vs. 29.6	2.2	0.68-6.96	0.186	25.0 vs. 9.5	3.2	0.50-20.0	0.371	29.4 vs. 17.4	2.0	0.44-8.87	0.456	14.3 vs. 9.5	1.6	0.20-12.8	1.000

Supplementary table 3: Median overall survival in different subgroups of patients. # cholangiocarcinoma was counted as low risk. ULN: upper limit of normal

	**Predictor**	**Variable**	median overall survival, years	p
Patients	Age	> 65 years vs. < 65 years	1.17 vs. 1.46	0.130
	BMI	> 30 kg/m2 vs < 30 kg/m2	1.04 vs. 1.44	0.437
Blood Biomarkers	CA 19-9	> median vs. < median	0.99 vs. 1.61	*0.040*
	CEA	> ULN vs. normal range	0.75 vs. 1.01	0.955
	Leucocyte count	> ULN vs. normal range	0.72 vs. 1.46	0.110
	CRP	> ULN vs. normal range	1.04 vs. 2.42	*0.016*
	CRP	> median vs. < median	0.75 vs. 2.31	*0.006*
	Bilirubin	> ULN vs. normal range	1.04 vs. 1.46	0.287
Tumor	Localization	Extrahepatic vs. intrahepatic	1.20 vs. 1.61	0.145
	Grading	High grade vs. low grade	1.01 vs. 2.31	0.121
	Metastasis	Distant metastasis vs. no distant metastasis	0.85 vs. 1.52	*0.072*
	Stage	Stage III/IV vs. stage I/II	1.17 vs. 2.42	0.216
	Stage	Stage IV vs. stage I/II/III	0.85 vs. 1.52	0.130
	Vascular compression	Vascular compression vs. no vascular compression	1.05 vs. 1.46	0.999
Treatment	Operation	Operation vs. no operation	2.51 vs. 0.65	< 0.001
	Best supportive care	Best supportive care vs. any treatment	0.25 vs. 1.52	< 0.001
	Chemotherapy	Chemotherapy vs. no chemotherapy	2.31 vs. 1.00	0.022
	Chemotherapy / operation	Chemotherapy vs. chemotherapy and operation	0.59 vs. 2.96	< 0.001
RAM #	Khorana score	≥ 2 vs. 0-1	0.72 vs. 1.45	0.817
	ONKOTEV score	≥ 2 vs. 0-1	0.72 vs. 1.52	*0.021*
	Protecht Score	≥ 3 vs. 0-2 (all patients)	1.20 vs. 1.44	0.819
	Protecht score	≥ 3 vs. 0-2 (only patients with chemotherapy)	1.20 vs. 2.42	*0.075*
Thromboembolism	Thromboembolism	Thromboembolism vs. no thromboembolism	0.72 vs. 1.61	0.107
		VTE vs. no TE	1.01 vs. 1.61	0.932
		PVT vs. no TE	1.05 vs. 1.61	0.229
		ATE vs. no TE	0.50 vs. 1.61	< 0.001
